# Pedobarographic evaluation of five commonly used orthoses for the lower extremity

**DOI:** 10.1007/s00402-022-04729-2

**Published:** 2022-12-26

**Authors:** C. Ehrnthaller, K. Rellensmann, S. F. Baumbach, M. Wuehr, R. Schniepp, M. M. Saller, W. Böcker, Hans Polzer

**Affiliations:** 1 Department of Orthopaedics and Trauma Surgery, Musculoskeletal University Center Munich (MUM), University Hospital, LMU, Marchioninistr. 15, 81377 Munich, Germany; 2 German Center for Vertigo and Balance Disorders, University Hospital, LMU, Munich, Germany

**Keywords:** Plantar pressure, Orthotic device, Foot and ankle surgery, Pedobarography

## Abstract

**Introduction:**

Orthoses are designed to achieve immobilization or off-loading of certain regions of the foot. Yet, their off-loading capacity for the specific regions has not yet been studied. Therefore, the aim of this study was to analyze the plantar pressure distribution of five commonly applied orthoses for foot and ankle in a healthy population.

**Materials and Methods:**

Five orthoses (postoperative shoe, forefoot relief shoe, short walker boot, high walker boot, and calcaneus fracture orthosis) were compared pedobarographically using insoles on a treadmill to a ready-made running shoe in eleven healthy subjects (median age 29 years). Peak pressure, maximum force, force–time integral, contact time, and contact area were evaluated separately for the forefoot, midfoot, and hindfoot.

**Results:**

The forefoot relief shoe, the short- and high walker boot significantly reduced the peak pressure at the forefoot with no significant differences between these orthoses. None of the five orthoses off-loaded the midfoot, but the calcaneus fracture orthosis and the short walker boot instead increased midfoot load. For the hindfoot, the calcaneus fracture orthosis was the only device to significantly reduce the peak pressure.

**Conclusions:**

This is the first study to investigate the specific off-loading capacities of different orthoses for specific foot regions in a healthy collective. The knowledge of absolute and relative load shifts for the different orthoses is of fundamental interest for targeted clinical decision-making of physicians.

**Supplementary Information:**

The online version contains supplementary material available at 10.1007/s00402-022-04729-2.

## Introduction

Orthotics are defined as ‘externally applied devices used to compensate for impairments of the structure and function of the neuro-muscular and skeletal systems’ (ISO 8549-1:2020, 3.1.2). General functions include immobilization, protected range of motion, selective load reduction, or the correction of the shape and function of the body. Thereby, they allow for earlier mobilization, pain reduction, and secure non-operative or operative treatment [1–3].

Orthoses are a frequently applied medical devices. About ten percent of the German population will use one at some point in their life [2]. One field in which orthoses are commonly applied is the foot and ankle, for example, to immobilize the ankle following arthrodesis [4], to unload the hindfoot in calcaneus fractures [5; 6], or to reduce mid- and forefoot forces after hallux valgus surgery [7, 8].

Despite their importance in foot and ankle rehabilitation, the number of studies investigating the selective load reduction of common orthoses within the foot is limited. Various studies have assessed the off-loading capacity of orthoses for the treatment of plantar ulcers in diabetic patients [9–11]. However, diabetes-associated comorbidities, such as peripheral neuropathy, postural impairments, and altered gait patterns, limit the translation of these results to the general population [12]. Other studies were limited to specific injuries, such as fractures to the fifth metatarsal bone [13], or anatomical locations, such as the hindfoot [14] or forefoot [15]. Consequently, up to date, no study has investigated the off-loading capacity of different types of orthoses for the different regions of the foot.

Therefore, the aim of the present study was to analyze the plantar pressure distribution of five common orthoses for the foot and ankle in a healthy population.

## Materials and methods

The herein presented study is a laboratory study and was approved by the local Ethics Committee (#18-882).

### Participants

Eleven healthy participants were recruited. Eligibility criteria were age between 18 and 65 years. Exclusion criteria were previous foot and ankle injuries/pathologies, pain during physical activities, peripheral vascular or neurological diseases, balance deficiencies, pregnancy or inability to provide informed consent.

### Orthoses

**Fig. 1:**
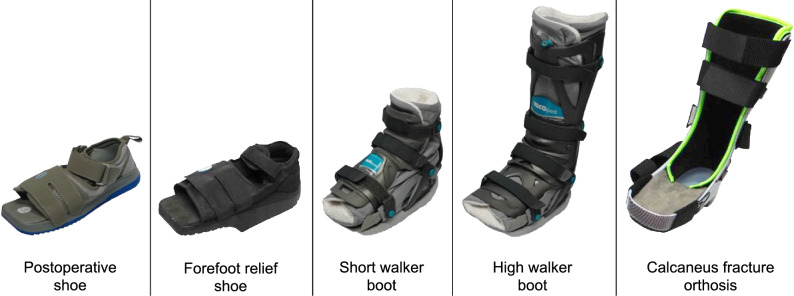
Illustration of the five orthoses analysed. Postoperative shoe: Relief Dual®; Darco; Forefoot relief shoe: OrthoWedge®; Darco; Short walker boot: VACOpedes®; Oped; High walker boot: VACOped®; Oped; Calcaneus fracture orthosis: CFO.®; perpedes)

Five commonly used foot and ankle orthoses were selected (Fig. 1). The postoperative shoe (Relief Dual®; Darco; USA) aims to reduce the pressure of the metatarsal region [16]. The forefoot relief shoe (OrthoWedge®; Darco; USA) has a step-shaped sole to eliminated forces to the forefoot [17]. The short walker boot (VACOpedes®; Oped; Germany) comprises a plastic shell with an inner vacuum cushion and is frequently applied for mid- and forefoot injuries. The high walker boot (VACOped®; Oped; Germany) is the high-cut version of the short walker boot, immobilizing the ankle, and is thought to reduce plantar pressure by redistribution [18]. It is commonly used, e.g., following Achilles tendon ruptures [19] and ankle fractures [20, 21]. The calcaneus fracture orthosis (CFO®; perpedes; Germany) consists of a polyethylene frame designed to unload the calcaneus and is used for the early functional treatment of calcaneus fractures [22].

Additionally, for the high walker boot the effect of a contralateral orthotic shoe lift was evaluated. The orthotic shoe lift (EVENUp®, Oped; Germany) is a synthetic sole of 1.27 cm height that is attached to the contralateral foot by elastic straps. It aims at leveling the leg length discrepancy [23].

### Insoles

The plantar pressure distribution was assessed using paroTec insoles (Paromed GmbH&Co.Kg, Neubeuern, Germany). They utilize 32 piezoresistive sensors with a silicon membrane imbedded in a hydro cell. They record compression as well as shear forces at a sample rate of 100 Hz. The pressure detection ranges are 0–700 kPa with a sensitivity of 0.51–0.66 mV/kPa and a reproducibility of ± 0.1% of the full scale. paroTec insoles exhibit a high reproducibility of 0.96 [24] and are frequently used in studies analyzing plantar pressure distribution [25, 26]. The insoles are available in six different sizes, and the appropriate size was chosen for each participant individually.

### Data acquisition

First, to determine the individual preferred walking speed [m/s], volunteers performed a 10-m walk in running shoes at a self-chosen comfortable pace. All orthoses were tested on the participant’s dominant leg and a running shoe on the supporting leg. The different orthoses were assessed in a standardized order: running shoe (control), postoperative shoe, forefoot relief shoe, short walker boot, high walker boot (ankle fixed in neutral position; with and without the orthotic shoe lift), and the calcaneus fracture orthosis. The appropriate insole was placed in the respective orthoses.

Prior to each recording, participants were allowed to walk in each orthosis until they felt comfortable. After static calibration of the insoles, the participants were asked to walk on a treadmill at their individual comfortable walking speed. After an initial adaption phase of 15 s, the pedobarographic data were recorded for 60 s.

### Data analysis

**Fig. 2:**
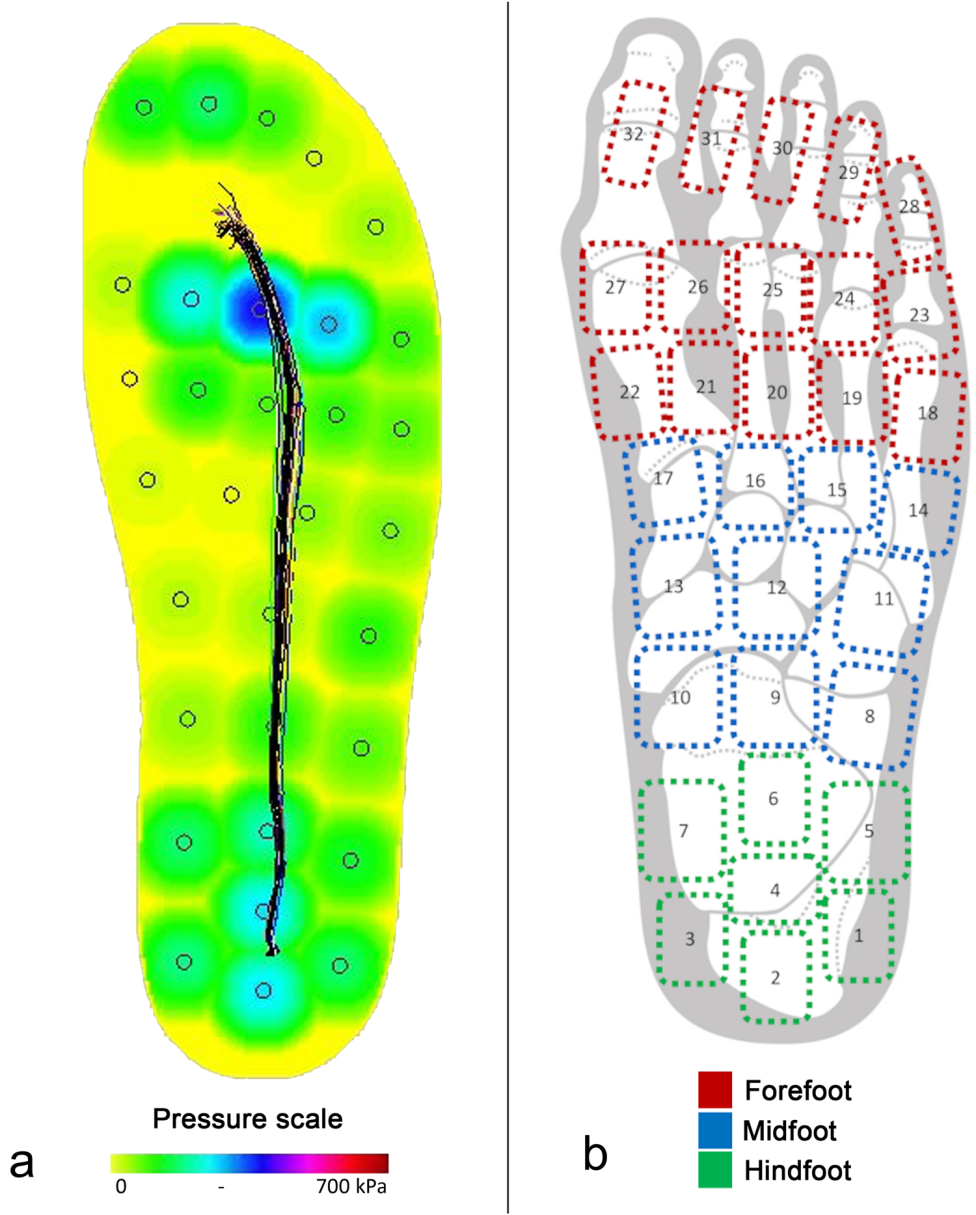
Illustration of the dynamic pressure measurements (running shoe) by the paroTec software (Paromed GmbH&Co.KG); black lines in a: trajectory of the center of pressure (**a**) and the sensor allocation into three foot regions (**b**)

A visual example of the paroTec recordings is illustrated in Fig. 2a. Each recording was exported from the paroTec software (Paromed GmbH&Co.KG, Neubeuern, Germany) for subsequent analysis in MATLAB (Matlab 2020b, The Mathworks, Natwick, USA). First, the plantar pressure data of the 32 sensors were subdivided into individual steps, i.e., successive periods of ground contact (48 ± 6 steps (mean ± SD), minimum 34 steps). Furthermore, the 32 sensors were divided into three different regions using an adaptation of the definition by Westphal et al. [27], the hindfoot (0–30% length), midfoot (31–60% length), and forefoot (61–100% length; Fig. 2b). Then, the following parameters (according to Nagel et al. [11]) were calculated for each individual step and region of interest: peak pressure (i.e., the highest local load), contact time (i.e., the duration of ground contact), contact area (i.e., the percentage of sensors detecting a load during ground contact), force time integral (the product of the amplitude and duration of force application), and maximum force. Finally, parameter estimates were averaged across all steps of a recording.

### Outcome parameters

The primary outcome parameter was the peak pressure [kPa]. The absolute values, as well as the relative values (% to normal loading of a running shoe), were calculated and analyzed. Secondary outcome parameters were the contact area [%], contact time [ms], force time integral [Ns], and the maximum force [N] for each orthosis. These were again analyzed as absolute and relative values. Finally, the effect of the orthotic shoe lift was evaluated by comparing absolute and relative values.

### Statistical analysis

The Shapiro–Wilk and Levene tests as well as QQ-plots were calculated in between the orthoses and foot regions and revealed no normal distribution. Therefore, nonparametric testing using the Wilcoxon test followed by a *p* value adjustment via Holm’s method was performed. *p* values < 0.05 were considered as statistically significant. The values are presented as median and interquartile range. The statistical analysis was performed in R (version 4.1.2 (2021-11-01)).

## Results

Eleven healthy volunteers (6 females) with a median age of 29 (range 26–38) years, were included. Their median BMI was 22 kg/m^2^ (range 17.9–25.5), and the median shoe size was 40 EU (range 37–46). The dominant leg was the left leg in six, and the right leg in five participants. The absolute and relative values for all pedobarographic parameters are presented in Supplementary Table 1.

### Primary outcome—peak pressure

**Fig. 3:**
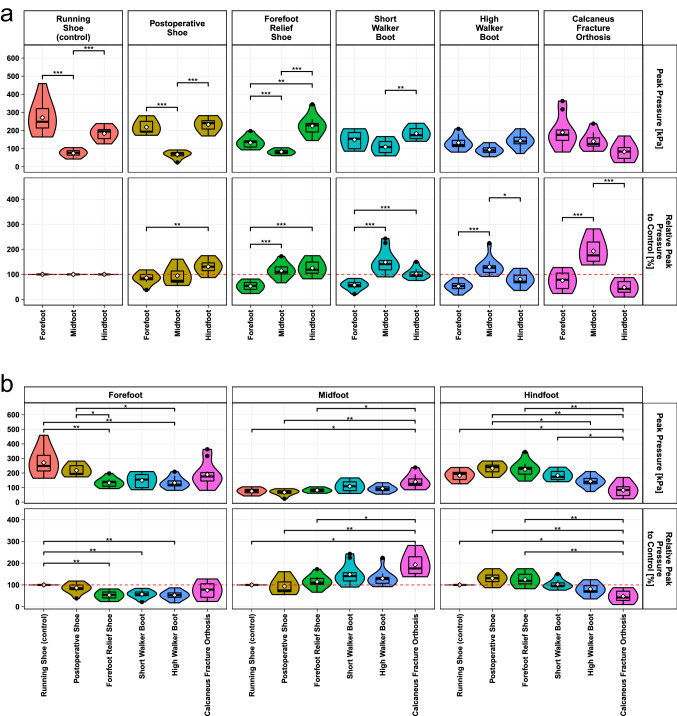
**a** Violin plots (representing the distribution of the measured values in a density-dependent manner) of the absolute peak pressure (upper) and the relative peak pressure values referenced to the running shoe (lower) comparing the different regions within each orthosis. **b** Violin plots of the absolute peak pressure (upper) and the relative peak pressure values referenced to the running shoe (lower) comparing the different orthoses for each foot region. *p < 0.05; **p < 0.01; ***p < 0.001; dashed line lower display (**a**, **b**) baseline values in running shoes represent 100%

The peak pressure values for each individual orthosis are presented in Fig. 3a. Every orthosis exhibited a specific peak pressure pattern with significant peak pressure differences between the three foot regions. The peak pressure values of each orthosis were compared in-between the three foot regions (Fig. 3b). For the forefoot, significantly lower absolute and relative peak pressure values were observed for the forefoot relief shoe and the high walker boot and lower relative peak pressure values for the short walker boot, when compared to the control. None of the orthoses significantly reduced the peak pressure of the midfoot. The calcaneus fracture orthosis showed significantly higher absolute and relative peak pressure values when compared to the postoperative shoe, forefoot relief shoe, and running shoe. For the hindfoot, the calcaneus fracture orthosis was the only one to significantly reduce the absolute and relative hindfoot peak pressures. Absolute values were significantly lower compared to all other orthoses but the high walker boot.

### Secondary outcomes

**Fig. 4:**
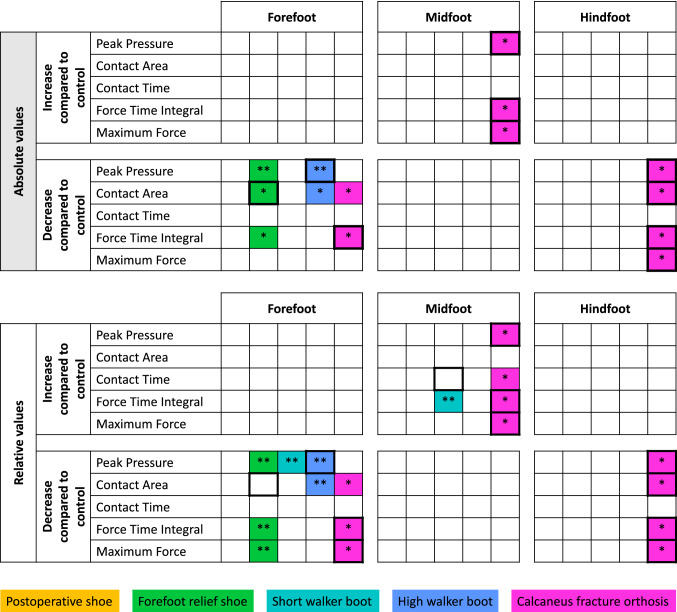
Summary of significant findings of primary and secondary outcome parameters between the orthoses, when compared to the control (running shoe). *p < 0.05; **p < 0.01; Dark frames: highest/lowest values

The absolute/relative values for the secondary outcome parameters, i.e., contact area, contact time, force time integral, and maximum force, are presented in the Supplementary Table 1. A summarizing illustration of the absolute and relative pedobarographic measures (including peak pressure) comparing the different orthosis within the three different foot regions is presented in Fig. 4.

For the forefoot, none of the assessed orthoses resulted in an increase of any parameter, when compared to the control. Out of all assessed orthoses, the high walker boot revealed the lowest peak pressure values (120 kPa (IQR: 34 kPa), the forefoot relief shoe the smallest contact area (73% (IQR: 14%), and the calcaneus fracture orthosis the lowest force time integral (47 Ns (IQR: 25 Ns).

For the midfoot, no orthosis showed a significant decrease of any of the assessed parameters. The calcaneus fracture orthosis was the only orthosis to increase the peak pressure (126 kPa (IQR: 45 kPa), force time integral (114 Ns (IQR: 74 Ns), and maximum force (272 N (IQR: 182 N) within the midfoot. The short walker boot did increase the relative force time integral significantly (181% (IQR: 119%).

For the hindfoot, no orthosis showed an increase of the assessed parameters. The calcaneus fracture orthosis decreased the peak pressure (84 kPa (IQR: 60 kPa), contact area (57% (IQR: 11%), force time integral (10 Ns (IQR: 16 Ns), and the maximum force (63 N (IQR: 71 N).

Finally, the effect of a contralateral orthotic shoe-lift to compensate for the resulting leg length discrepancy when wearing a high walker boot was assessed. Comparing absolute and relative values for all primary and secondary outcome parameters with versus without orthotic shoe lift did not reveal any significant differences.

## Discussion

This is the first study to assess the off-loading capacity for specific foot regions of five commonly used orthoses in foot and ankle surgery. Regarding the forefoot, the forefoot relief shoe, the short and high walker boot showed a comparable off-loading capacity. None of the assessed orthoses was capable of off-loading the midfoot, but the calcaneus fracture orthosis and the short walker boot instead increased the load within the midfoot. The calcaneus fracture orthosis was the only device to significantly off-load the hindfoot.

Orthotic devices are used to off-load specific foot regions with the aim to enable earlier mobilization, pain reduction and to secure successful treatment [1, 2, 28]. Despite their frequent use, information regarding the specific off-loading capacity of commonly used orthoses is sparse.

The forefoot relief shoe, short and high walker boot significantly reduced the plantar pressure in the forefoot compared to a running shoe. These findings are in line with previous studies, which reported an equal off-loading effect of the forefoot for vacuum orthoses, such as walker boots, and cushioning orthotic devices, such as forefoot relief shoes [9, 11]. Interestingly, the short walker boot only yielded significant reductions in relative peak pressure values, but did neither affect absolute peak pressure values nor any other assessed pedobarographic parameter. In contrast, the forefoot relief shoe, high walker boot, and calcaneus fracture orthosis significantly reduced absolute and relative values for two or more parameters.

None of the assessed orthotic devices generated a significant pressure reduction in the midfoot. Consequently, none of these orthoses appears to be appropriate for applications, in which an off-loading of the midfoot is aspired. Therefore, if pressure in the midfoot region is to be reduced, an orthosis should be combined with partial-weightbearing. Controversy, in particular the calcaneus fracture orthosis significantly increased the midfoot load, which has been reported previously [14]. This load shift toward the midfoot may lead to secondary displacement in calcaneus fractures affecting the anterior process of the calcaneus, cause pain, or stress fractures in case of long-term use [14]. It might also explain why the calcaneus fracture orthosis is often doomed uncomfortable by patients.

Mazur et al. [14] compared pedobarographic measurements between a running shoe and two different hindfoot relief orthoses—the hindfoot relief shoe and the hindfoot relief orthosis in 25 healthy volunteers. They reported an off-loading capacity for the hindfoot of 52% for the two tested devices, which is in line with the herein observed 57% for the calcaneus fracture orthosis.

Finally, we also assessed the effect of a contralateral shoe lift when using a high walker boot on the plantar pressure characteristics. Palmanovich et al. [29] investigated the load effect of a contralateral leg-length equalizing sole when wearing a forefoot relief shoe in 20 healthy men. Similar to our presented data, they could not detect any significant changes in peak pressure and peak pressure integral. However, previous studies were able to show a reduction of gait asymmetries [30], significant improvements in the modified Oswestry low back pain disability questionnaire, and higher scores in the lower extremity functional scale [23] when an orthotic shoe lift was used.

One limitation of the present study is that only the orthosis-specific plantar pressure distribution was assessed, but not the ability of immobilization. Some orthoses, especially the high walker boot, have fields of application due to their ability to immobilize the hind- and midfoot [31], that go beyond the plantar off-loading. This aspect was not addressed in the present study. Furthermore, we retained the same sequence in testing the orthoses for each participant. This holds a potential bias. Another limitation is the rather small sample size and the focus on healthy individuals. Patients suffering from pain and/or gait insecurity might exhibit considerably different patterns of plantar pressure distribution. Therefore, it remains unclear, whether the present observations can be generalized to a clinical setting. Despite these limitations, this study established a standardized assessment setting that not only focused on the absolute effects of orthoses on specific pedobarographic measures, but also analyzed their relative effect in comparison with a control shoe. Any orthosis must proof its off-loading superiority to a regular shoe. By standardizing our measurements to a control, i.e., running shoe, we were able to highlight this real-life advantage of the respective orthosis.

## Conclusion

The results provide new insights into the efficiency of five commonly used orthoses to modify pressure distribution patterns in specific foot regions. The knowledge of the specific pressure distribution of the different orthoses is essential for orthopedics to specifically choose the appropriate device. Therefore, the clinical use of specific off-loading orthoses should be indicated carefully and critically discussed as they might induce the risk to increase pressure distribution at the adjoining foot regions. These results should further be validated in larger healthy and patient cohorts.

## Supplementary Information

Below is the link to the electronic supplementary material.

Supplementary file1 (DOCX 35 KB)
