# Supplementary material for: Pedobarographic evaluation of five commonly used orthoses for the lower extremity
Source: Arch Orthop Trauma Surg. 2022 Dec 26;143(7):4249–56. doi: 10.1007/s00402-022-04729-2 (PMC10293377; doi:10.1007/s00402-022-04729-2)
Supplement: Supplementary file 1 — Supplementary file1 (DOCX 35 KB) [file 402_2022_4729_MOESM1_ESM.docx]

|  |  |  |  | **Forefoot** | |  | **Midfoot** | |  | **Hindfoot** | |
| --- | --- | --- | --- | --- | --- | --- | --- | --- | --- | --- | --- |
|  |  |  |  | median | IQR |  | median | IQR |  | median | IQR |
| Peak Pressure [kPa] |  | **Running shoe (control)** |  | 248 | 107 |  | 77 | 24 |  | 195 | 50 |
|  |  | **Postoperative shoe** |  | 194 | 59 |  | 72 | 18 |  | 241 | 46 |
|  |  | **Forefoot relief shoe** |  | 137 | 34 |  | 80 | 18 |  | 229 | 47 |
|  |  | **Short walker boot** |  | 155 | 90 |  | 109 | 61 |  | 174 | 55 |
|  |  | **High walker boot** |  | 120 | 34 |  | 90 | 25 |  | 140 | 37 |
|  |  | **Calcaneus fracture orthosis** |  | 176 | 57 |  | 126 | 45 |  | 84 | 60 |
|  |  |  |  |  |  |  |  |  |  |  |  |
| Contact Area  [%] |  | **Running shoe (control)** |  | 100 | 0 |  | 97 | 25 |  | 100 | 0 |
|  |  | **Postoperative shoe** |  | 99 | 4 |  | 65 | 18 |  | 100 | 0 |
|  |  | **Forefoot relief shoe** |  | 73 | 14 |  | 79 | 12 |  | 100 | 0 |
|  |  | **Short walker boot** |  | 93 | 19 |  | 100 | 4 |  | 100 | 0 |
|  |  | **High walker boot** |  | 82 | 14 |  | 92 | 10 |  | 100 | 0 |
|  |  | **Calcaneus fracture orthosis** |  | 84 | 7 |  | 100 | 1 |  | 57 | 11 |
|  |  |  |  |  |  |  |  |  |  |  |  |
| Contact Time [ms] |  | **Running shoe (control)** |  | 668 | 51 |  | 623 | 118 |  | 520 | 107 |
|  |  | **Postoperative shoe** |  | 697 | 38 |  | 540 | 54 |  | 559 | 48 |
|  |  | **Forefoot relief shoe** |  | 626 | 48 |  | 617 | 35 |  | 625 | 52 |
|  |  | **Short walker boot** |  | 579 | 113 |  | 691 | 59 |  | 555 | 127 |
|  |  | **High walker boot** |  | 651 | 51 |  | 670 | 46 |  | 622 | 108 |
|  |  | **Calcaneus fracture orthosis** |  | 593 | 190 |  | 710 | 29 |  | 404 | 245 |
|  |  |  |  |  |  |  |  |  |  |  |  |
| Force Time Integral [Ns] |  | **Running shoe (control)** |  | 115 | 43 |  | 51 | 29 |  | 82 | 19 |
|  |  | **Postoperative shoe** |  | 130 | 38 |  | 32 | 20 |  | 86 | 14 |
|  |  | **Forefoot relief shoe** |  | 62 | 20 |  | 48 | 25 |  | 90 | 25 |
|  |  | **Short walker boot** |  | 73 | 50 |  | 92 | 34 |  | 66 | 14 |
|  |  | **High walker boot** |  | 59 | 29 |  | 78 | 30 |  | 58 | 22 |
|  |  | **Calcaneus fracture orthosis** |  | 47 | 25 |  | 114 | 74 |  | 10 | 16 |
|  |  |  |  |  |  |  |  |  |  |  |  |
| Maximum Force [N] |  | **Running shoe (control)** |  | 407 | 102 |  | 128 | 76 |  | 329 | 128 |
|  |  | **Postoperative shoe** |  | 387 | 103 |  | 94 | 56 |  | 299 | 173 |
|  |  | **Forefoot relief shoe** |  | 221 | 59 |  | 124 | 65 |  | 294 | 123 |
|  |  | **Short walker boot** |  | 265 | 178 |  | 227 | 65 |  | 279 | 86 |
|  |  | **High walker boot** |  | 220 | 82 |  | 179 | 43 |  | 199 | 59 |
|  |  | **Calcaneus fracture orthosis** |  | 194 | 52 |  | 272 | 182 |  | 63 | 71 |

|  |  |  |  | **Forefoot** | |  | **Midfoot** | |  | **Hindfoot** | |
| --- | --- | --- | --- | --- | --- | --- | --- | --- | --- | --- | --- |
|  |  |  |  | median | IQR |  | median | IQR |  | median | IQR |
| Relative  Peak Pressure  [%] |  | **Running shoe (control)** |  | 100 | 0 |  | 100 | 0 |  | 100 | 0 |
|  |  | **Postoperative shoe** |  | 84 | 21 |  | 75 | 45 |  | 131 | 31 |
|  |  | **Forefoot relief shoe** |  | 54 | 27 |  | 110 | 28 |  | 119 | 44 |
|  |  | **Short walker boot** |  | 57 | 17 |  | 141 | 37 |  | 96 | 18 |
|  |  | **High walker boot** |  | 53 | 20 |  | 127 | 27 |  | 71 | 30 |
|  |  | **Calcaneus fracture orthosis** |  | 78 | 62 |  | 176 | 77 |  | 42 | 42 |
|  |  |  |  |  |  |  |  |  |  |  |  |
| Relative  Contact Area  [%] |  | **Running shoe (control)** |  | 100 | 0 |  | 100 | 0 |  | 100 | 0 |
|  |  | **Postoperative shoe** |  | 100 | 4 |  | 76 | 27 |  | 100 | 0 |
|  |  | **Forefoot relief shoe** |  | 73 | 14 |  | 95 | 21 |  | 100 | 0 |
|  |  | **Short walker boot** |  | 93 | 18 |  | 103 | 22 |  | 100 | 0 |
|  |  | **High walker boot** |  | 83 | 13 |  | 101 | 27 |  | 100 | 0 |
|  |  | **Calcaneus fracture orthosis** |  | 84 | 8 |  | 113 | 33 |  | 57 | 11 |
|  |  |  |  |  |  |  |  |  |  |  |  |
| Relative  Contact Time  [%] |  | **Running shoe (control)** |  | 100 | 0 |  | 100 | 0 |  | 100 | 0 |
|  |  | **Postoperative shoe** |  | 99 | 7 |  | 85 | 17 |  | 111 | 14 |
|  |  | **Forefoot relief shoe** |  | 94 | 6 |  | 99 | 16 |  | 126 | 33 |
|  |  | **Short walker boot** |  | 93 | 17 |  | 114 | 17 |  | 116 | 35 |
|  |  | **High walker boot** |  | 94 | 7 |  | 111 | 24 |  | 117 | 31 |
|  |  | **Calcaneus fracture orthosis** |  | 92 | 27 |  | 108 | 12 |  | 75 | 44 |
|  |  |  |  |  |  |  |  |  |  |  |  |
| Relative  Force Time Integral  [%] |  | **Running shoe (control)** |  | 100 | 0 |  | 100 | 0 |  | 100 | 0 |
|  |  | **Postoperative shoe** |  | 98 | 16 |  | 68 | 47 |  | 113 | 21 |
|  |  | **Forefoot relief shoe** |  | 59 | 17 |  | 145 | 82 |  | 119 | 58 |
|  |  | **Short walker boot** |  | 51 | 28 |  | 181 | 119 |  | 82 | 34 |
|  |  | **High walker boot** |  | 46 | 13 |  | 170 | 73 |  | 74 | 50 |
|  |  | **Calcaneus fracture orthosis** |  | 43 | 27 |  | 282 | 206 |  | 12 | 22 |
|  |  |  |  |  |  |  |  |  |  |  |  |
| Relative  Maximum Force  [%] |  | **Running shoe (control)** |  | 100 | 0 |  | 100 | 0 |  | 100 | 0 |
|  |  | **Postoperative shoe** |  | 96 | 14 |  | 62 | 49 |  | 110 | 20 |
|  |  | **Forefoot relief shoe** |  | 57 | 17 |  | 128 | 50 |  | 102 | 42 |
|  |  | **Short walker boot** |  | 65 | 22 |  | 156 | 95 |  | 76 | 15 |
|  |  | **High walker boot** |  | 52 | 14 |  | 152 | 71 |  | 74 | 33 |
|  |  | **Calcaneus fracture orthosis** |  | 46 | 23 |  | 233 | 130 |  | 21 | 25 |
